# Current distribution monitoring enables quench and damage detection in superconducting fusion magnets

**DOI:** 10.1038/s41598-022-26592-2

**Published:** 2022-12-28

**Authors:** Reed Teyber, Jeremy Weiss, Maxim Marchevsky, Soren Prestemon, Danko van der Laan

**Affiliations:** 1 Lawrence Berkeley National Laboratory, Berkeley, CA 94720 USA; 2 Advanced Conductor Technologies, Boulder, CO 80309 USA; 3 Department of Physics, University of Colorado, Boulder, CO 80309 USA

**Keywords:** Applied physics, Techniques and instrumentation

## Abstract

Fusion magnets made from high temperature superconducting ReBCO CORC^®^ cables are typically protected with quench detection systems that use voltage or temperature measurements to trigger current extraction processes. Although small coils with low inductances have been demonstrated, magnet protection remains a challenge and magnets are typically operated with little knowledge of the intrinsic performance parameters. We propose a protection framework based on current distribution monitoring in fusion cables with limited inter-cable current sharing. By employing inverse Biot-Savart techniques to distributed Hall probe arrays around CORC^®^ Cable-In-Conduit-Conductor (CICC) terminations, individual cable currents are recreated and used to extract the parameters of a predictive model. These parameters are shown to be of value for detecting conductor damage and defining safe magnet operating limits. The trained model is then used to predict cable current distributions in real-time, and departures between predictions and inverse Biot-Savart recreated current distributions are used to generate quench triggers. The methodology shows promise for quality control, operational planning and real-time quench detection in bundled CORC^®^ cables for compact fusion reactors.

## Introduction

ReBCO cables are an enabling technology for compact fusion reactors^1–3^, due to the high critical temperature, high critical field and potential to form demountable magnets^4^. Commonwealth Fusion Systems are developing compact fusion reactors^5^ based on their VIPER ReBCO cable design^6^. Tokamak Energy is also developing compact fusion reactors^7^ based on ReBCO conductors. A magnet concept for the Fusion Nuclear Science Facility (FNSF) was recently proposed based on CORC^®^ Cable-In-Conduit-Conductors (CICC)^8^ that consist of transposed CORC^®^ cables^9^ around a former in a 6-around-1-like structure^10,11^. A CORC^®^ solenoid was recently tested in a 14 T background field^12^ in an effort to prove the high-field capabilities of the conductor. With similar research objectives, a separate CORC^®^-like solenoid was tested in a 19 T background field for the China Fusion Engineering Test Reactor (CFETR)^13^.

Although rapid progress is being made toward ReBCO cables for fusion reactors, quench detection and magnet protection remain an active area of research^2,14,15^. In tokamaks, the large magnet inductances and fast ramp rates complicate protection with traditional sample voltage measurements. Several efforts have shifted toward temperature-based protection, including optical fibers^16–19^, active acoustic thermometry^20^, and co-wound superconducting wires optimized to act as a thermal switch^21–23^. Marchevsky *et al.*^24^ demonstrated quench detection in a slit ReBCO tape based on magnetic field changes associated with current redistribution, that was later demonstrated between tapes of a single CORC^®^ wire^25^ and between cables of a CORC^®^ CICC^26^. These Hall probe-based techniques can be highly sensitive to events that precede thermal runaway; however, the phenomena underlying the magnetic field measurements should be resolved to make informed decisions in real time. With a similar motivation, a recent study combined Hall sensors and sample voltages to monitor transient non-insulated ReBCO coils in real time^27^.

**Figure 1:**
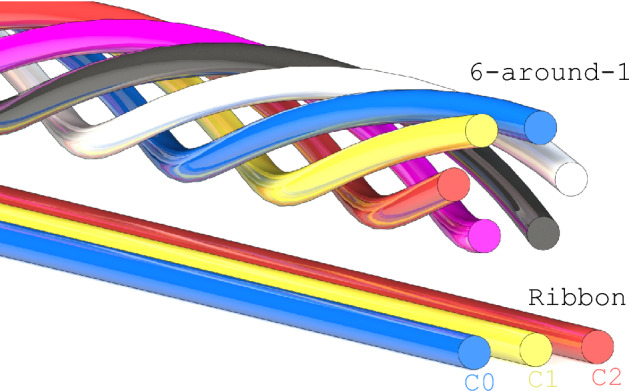
Ribbon CICC (bottom) and 6-around-1 (top) wound with CORC^®^ cables. Both are high-current cables relevant for fusion with limited current sharing between sub-elements.

While these references describe technology development, there is still a need to demonstrate robust ReBCO magnet protection under the demanding conditions found in fusion reactors; this may require a complimentary portfolio of diagnostics. In this work we propose and develop a framework around current distribution monitoring in fusion cables with limited inter-cable current sharing, such as the 6-around-1 shown in Fig. 1, to supplement voltage and temperature-based protection. Measurements with distributed Hall probe arrays are coupled with an inverse Biot-Savart process to recreate individual cable currents in real time. This allows the parameters of a dynamic network model to be extracted, including the distribution of termination resistances and cable critical currents. This can be used to identify a poor joint or conductor damage; this is of importance for quality control in fusion magnets. Using the previously published data of Weiss et al.^26^, the trained model is then used to predict CICC current distributions, and departures between predictions and inverse Biot-Savart recreated current distributions are used to generate quench triggers.

The manuscript is organized as follows. First, the methodology is outlined which includes a description of CORC^®^ fusion cables, dynamic network modeling, calculation of the inductance matrix and the inverse Biot-Savart technique. The process of combining these aspects into a data-driven diagnostic for quench detection is then presented. This is followed by the results section which describes the parameter extraction process followed by simulations using real cable parameters. Finally, the quench detection capabilities are presented using the previously published three-cable Ribbon CICC of Ref.^26^ at 2000 A/s.

## Methods

### CICC network model

**Figure 2:**
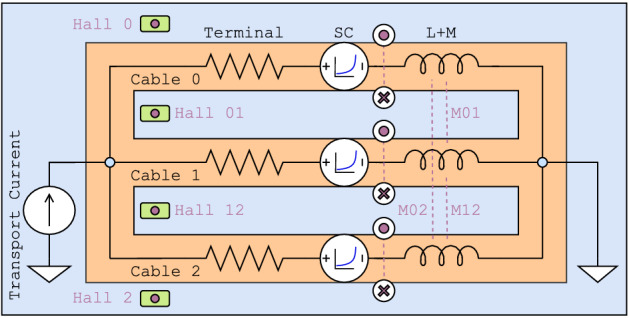
Circuit diagram for the CORC^®^ CICC triplet of Ref.^26^. Hall probe positioning and field distribution are shown in Fig. 4. Purple arrows and circles show the direction of magnetic field produced by the current in each cable branch.

The methodology relies on the ability to predict current distributions in CICC with limited inter-cable current sharing, that applies to CORC^®^ CICC concepts such as the ribbon CICC^26^ and 6-around-1^10^ shown in Fig. 1. The focus of this work is the three-cable Ribbon CICC (i.e., triplet) of Ref.^26^. Although the 6-around-1 is symmetric, the Ribbon CICC has asymmetries in the inductance matrix that can lead to current maldistribution during ramping. The circuit diagram for the three-cable CORC^®^ CICC of Ref.^26^ is shown in Fig. 2. Each cable consists of a termination resistance (both terminal resistances together), a single superconductor exhibiting a current-voltage (I-V) transition (entire length of all tapes lumped into a single superconductor) and an inductor that is globally connected. There is no current sharing between cables, and four Hall probes are illustrated near the terminations.

The inductance is evaluated with the Neumann integral, where each superconducting sub-element is simplified as a line current with no consideration of a critical state model or magnetization.

1 $$\begin{aligned} \begin{aligned} L_{ij} = \frac{\mu _0}{4\pi } \int \int \frac{\vec {d\textbf{x}_i}\cdot \vec {d\textbf{x}_j}}{|\vec {\textbf{x}_i} - \vec {\textbf{x}_j}|} \end{aligned} \end{aligned}$$

The self inductance $L_{ii}$ is calculated in a similar way, however the integral between any points closer than half the cable radius are not considered and a correction term is added of $\mu _0 l_{cable} / 8\pi$^28,29^. Although these are simplifying approximations of CORC^®^ inductance, general behaviours and sensitivities are captured and errors are reduced using the semi-analytic approach described below.

### Inverse Biot-Savart current recreation

**Figure 3:**
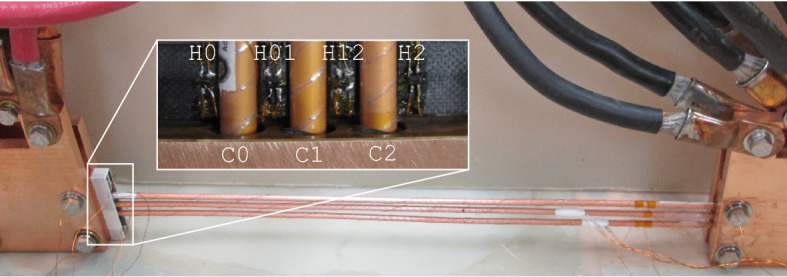
Experimental setup from Weiss et al.^26^ with cable and Hall probe labels corresponding to Fig. 4.

**Figure 4:**
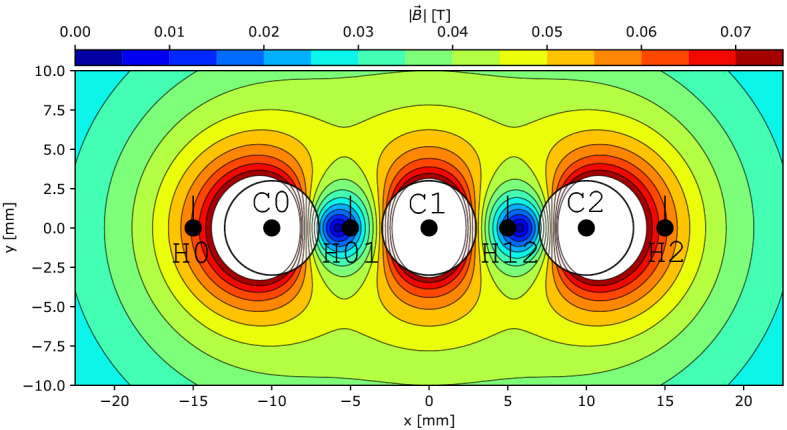
Geometry and Biot-Savart field calculation of CORC^®^ triplet CICC data in Ref.^26^ with 1 kA in each cable. CICC configuration corresponds to the network schematic in Fig. 2.

The CORC^®^ CICC configurations exhibit limited current sharing, forcing the majority of current to redistribute through terminations that can be monitored with Hall probe arrays. In contrast to individual cables that are electromagnetically complex, the fusion cable-of-cable configurations are well-approximated as line currents which reduce the number of unknowns in an inverse process to recreate current distributions. A current recreation process is developed here for the triplet experiments of Ref.^26^, shown in Figs. 3 and  4, consisting of three non-transposed, 0.5 meter long CORC^®^ cables (x = − 10, 0 and 10 mm, y = 0 mm) and four single axis commercial GaAs Hall probes (x= − 15, − 5, 5 and 15 mm, y = 0 mm) oriented vertically along the *y* axis. More details on the sample, Hall probe instrumentation, measurements and test protocols can be found in Ref.^26^.

If a line current at cable *i* in the *z* direction (i.e., out of page) extends adequately far on either side of an ideal single-axis Hall probe *k*, the measured field is:

2 $$\begin{aligned} \begin{aligned} A_{ik}^* = \frac{\mu _0 }{2\pi |\vec {r}_{ik}|^2}<-r_{ik,y}, r_{ik,x}> \cdot <n_{k, x}, n_{k, y}> \\ B_{ik} = A_{ik}^*I_{i,z}\\ \end{aligned} \end{aligned}$$

that is the projection of the familiar $B=\mu _0I/2\pi r$ onto the Hall probe measurement axis $<n_{k, x}, n_{k, y}>$ (see Fig. 4). $B_{ik}$ is the field measured by the single-axis Hall probe *k* from the single current at cable *i*, $I_{i,z}$ is the current in the *z* direction of cable *i* and $\vec {r}_{ik}$ is the x–y vector between line current *i* and Hall probe *k*. The equation is split over two lines to highlight that $B_{ik}$ is a linear function of current. For terminations with more complex geometries, the differential Biot-Savart law can be integrated with unitary current. Following the implementation of Ref.^30^, in the absence of magnetic material and sensor positioning errors the measured response at Hall probe *k* is the sum of Eq. 2 over all $n_i$ cables:

3 $$\begin{aligned} B_{k} = \sum _{i}^{n_i} A_{ik}^*I_{i,z} \end{aligned}$$

Re-writing Eq. 3 for each of $n_k$ sensors yields a matrix system with a row for each Hall sensor and a column for each cable current; each entry in the matrix *A* consists of a cable-sensor pair.

4 $$\begin{aligned} \begin{bmatrix} A_{00}^* &{} A_{i0}^* &{} A_{n_i0}^* \\ A_{0k}^* &{} A_{ik}^* &{} A_{n_ik}^* \\ A_{0n_k}^* &{} A_{in_k}^* &{} A_{n_in_k}^* \\ \end{bmatrix} \begin{bmatrix} I_{0,z} \\ I_{i,z} \\ I_{n_i,z} \\ \end{bmatrix} = \begin{bmatrix} B_{0} \\ B_{k} \\ B_{n_k} \\ \end{bmatrix} \end{aligned}$$

If there are more Hall sensors than cable currents, as is the case for the triplet data in Ref.^26^ (four sensors for three currents, see Fig. 4), the least squares form is considered:

5 $$\begin{aligned} A^TAx=A^Tb \end{aligned}$$

This linear system $A^TAx=A^Tb$ can be solved to obtain the cable current distribution, and Ref.^30^ outlines several techniques to improve the stability of the dense and ill-conditioned system. Previously, a similar technique based on Singular Value Decomposition (SVD) was employed to investigate current distributions in ITER cables^31^, however the high levels of current sharing did not permit the methodology employed here. It is possible to solve the least squares problem with a net transport current constraint (see Ref.^30^), however this was not necessary here.

### Current redistribution based quench detection

**Figure 5:**
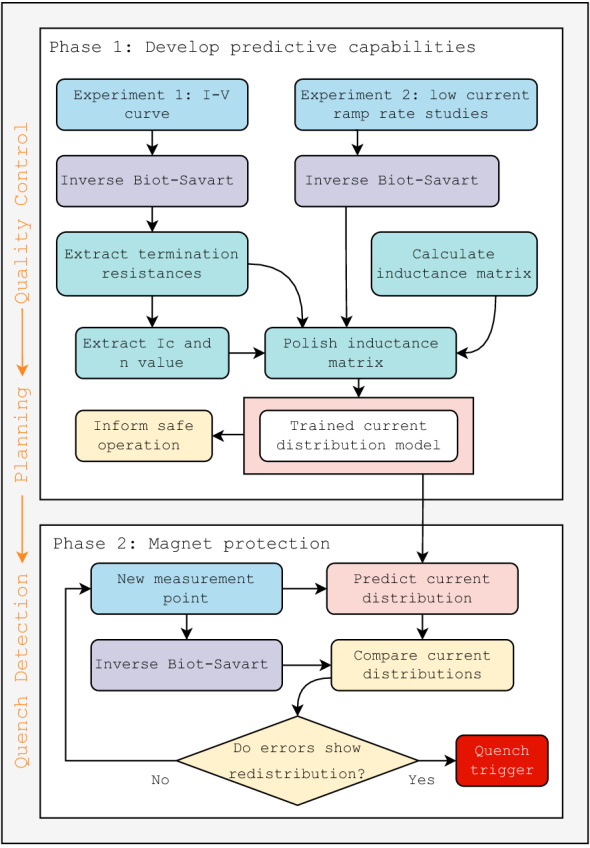
Methodology of this work. Cable currents are recreated from experiments using inverse Biot-Savart techniques, which are used to extract electric circuit parameters. These parameters can be used for quality control and test planning. The second phase compares the trained model predictions of cable currents with inverse Biot-Savart recreated cable currents for quench detection.

If the behavior of a CORC^®^ CICC can be predicted with a dynamic network model, and experimental current distributions can be recreated using the inverse Biot-Savart process, then it is possible to detect normal zones and protect fusion magnets by monitoring current distributions. The methodology proposed in this manuscript is outlined in Fig. 5, which exploits the ability to monitor current distributions at cable terminations. Phase one relies on extracting the electric circuit parameters shown in Fig. 2. The first step in phase one is to perform an I-V curve with distributed Hall probe measurements at the magnet operating temperature, where the test is performed conservatively. By recreating the current distributions, the distribution of termination resistances and critical currents in each cable can be extracted. For especially risk-adverse magnets, the I-V test can be performed first at 77 K and the methodology can inform protection during the first low temperature I-V curves. The ability to extract the distribution of termination resistances and critical currents serves a role in quality control, as outlined in orange on the left of Fig. 5, where a poor joint or damaged conductor can be addressed before more strenuous magnet operation.

This is followed by a second set of experiments aimed at characterizing the dynamic behavior, where the cable current distributions are recreated during a fast ramp. These ramp rate tests are performed conservatively at low currents, which assumes a linear inductance-current relationship. A purely data-driven extraction of the inductance matrix is challenging, and the line current treatment of CORC^®^ cables makes an analytic approach less robust. As such, a semi-analytic procedure is implemented where the analytic inductance matrix (Eq. 1) is polished to fit training data. In this process, small variations in the cable spacing and individual cable lengths are used as optimization variables in a least-squares fit to match spice simulations to selected low current ramp rate studies. By varying both the cable spacing and the cable lengths, the self and mutual inductances can be tuned. Although a large number of inductance integrations are required, perturbing the geometry instead of the resulting matrix entries avoids potential energy conservation issues. It should be emphasized that the training data (data used in the parameter extraction) is separated from the test data (quench detection results presented below); we do not fit the model to any tests used to demonstrate quench detection.

The result is a data-driven network model that has been tuned to the magnet being protected. This model can then be used to run planning cases and identify hazardous operating conditions. This trained model also serves as the groundwork for the magnet protection phase (phase 2 in Fig. 5). The quench detection scheme is based on real-time differences between recreated and model-predicted cable currents for each incoming measurement. Both the magnitude of error and the rate of change of error are monitored. The results presented here use error thresholds of 50 A and 250 A/s, however these will be specific to the magnet and current profile being tested. Although prediction errors will always exist, a rapid departure of model accuracy (i.e., error rate in A/s) is characteristic of current redistribution arising from a cable quench. In addition to a cable current decreasing until the error threshold is met, there must be an increase in current in the remaining cables. This criteria substantially decreases false positive quench signals, along with requiring the thresholds to be violated for a set number of measurements (five here).

## Results

### Parameter extraction and quality control

**Figure 6:**
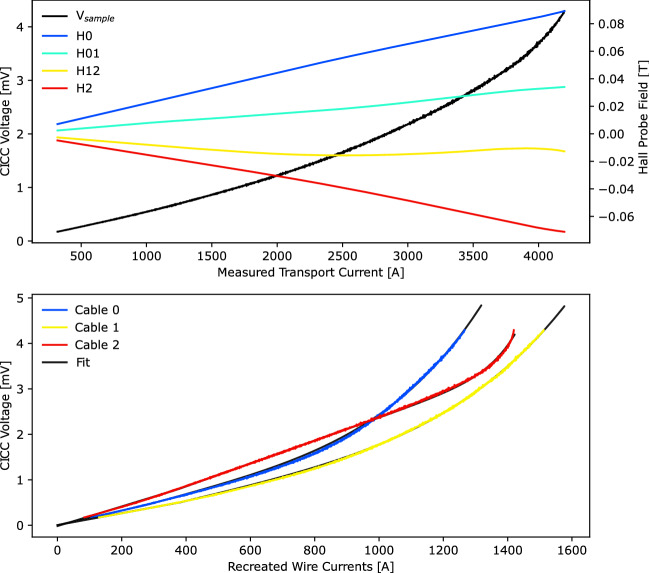
Processing of I-V curve with Hall probe measurements (top plot, see Fig. 4) into recreated cable currents with model fits (bottom plot).

The process in Fig. 5 of training a model is presented here using the previously published data of Weiss et al.^26^. The sample consists of three non-transposed cables in a ribbon CICC formation, with a cable spacing of 10 mm and a total CICC length of 0.5 m (see Fig. 3). The experimental dataset includes I-V curves, measurements at various ramp rates and heater-induced quenches at 76 K (liquid nitrogen bath in Boulder, Colorado). The top plot in Fig. 6 shows an I-V measurement, where the total sample voltage and four Hall probes in Fig. 4 are measured as a function of the transport current. This total sample voltage is upstream of the cables and consists of a resistive voltage from the copper bus.

The data in the top plot of Fig. 6 is processed into the data in the bottom plot; the total sample voltage (same across three parallel cables) is shown on the y axis as a function of the recreated current in each cable on the x axis. This disaggregates the individual cable I-V characteristics from the bulk CICC measurement, and allows all termination resistances and cable critical currents to be extracted from a single measurement. The curve fit is shown in black behind the colored lines and is summarized in Tables 1, 2. A piecewise linear (i.e., bi-linear) terminal resistance fit improved the prediction accuracy of these experiments. From a single I-V curve and the methodology presented thus far, cable damage from winding can be recognized and terminal resistance maldistribution can be identified. This information can be used to motivate repairs, and the parameter extraction can be repeated on a maintenance schedule to track changes in performance with time. Although the model training should be performed with experiments at the same magnet temperature as the intended operation, this quality control information can be obtained from a single 77 K I-V curve.

**Table 1:** Extracted termination resistances for triplet fit shown in Fig. 6.

Cable	$I_{thresh} [A]$	$R_{term}^{\le } [\upmu \Omega ]$	$R_{term}^{>} [\upmu \Omega ]$
Cable 0	320	1.63	1.93
Cable 1	377	1.30	1.64
Cable 2	313	2.07	2.50

A bi-linear fit is implemented, resulting in different resistances before ($\le$) and after (>) the threshold current $I_{thresh} [A]$. These resistances are based on the sample voltage, which is the upstream voltage of the cables and includes a substantial resistive voltage from the copper bus.

**Table 2:** Extracted critical current parameters for the triplet fit shown in Fig. 6, where a slow and early superconducting transition is seen in cables 0 and 1.

Cable	$I_{C} [A]$	$n [-]$
Cable 0	963	5.0
Cable 1	1146	4.8
Cable 2	1385	16.4

A 0.5 mV critical current criteria ($10 \upmu$ V/cm) is considered; this is less conservative than the typical $1 \upmu$ V/cm electric field criterion.

The next step is to characterize the dynamic behavior using the previously described semi-analytic treatment. The inductance matrix polishing is performed here using a single low-current 5000 A/s up-and-down ramp, and the resulting inductance matrix is shown in Table 3. For the short straight sample investigated here, the analytic calculation was acceptable and polishing made only a marginal improvement; however, polishing is expected to be required with longer, more complex winding geometries. The inductance polishing process here considers only the current distributions and not the measured sample voltage, as the measured sample voltage used in the termination resistance fit above does not consider the integrated resistance through cables and through the copper bus bars. In other words, induced current loops that flow through the terminations and copper bus bars and then back through CORC^®^ cables are not perfectly captured by the CICC sample voltage measurement, and hence the dynamic performance of the individual cables in the CICC can only be captured by fitting the current distributions.

**Table 3:** Semi-analytic inductance Matrix [$\upmu$ H] of the triplet CICC in Ref.^26^.

	C0	C1	C2
C0	0.53	0.37	0.29
C1		0.52	0.35
C2			0.51

### Simulated cable performance

**Figure 7:**
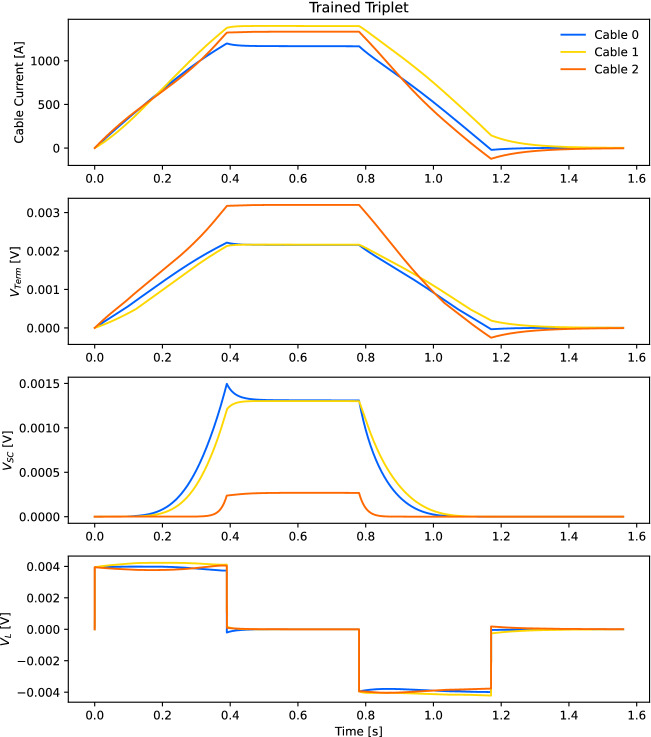
Simulated performance of the triplet cable during a fast trapezoidal ramp to 3900 A at 10,000 A/s using the extracted parameters in Tables 1, 2, 3. The top plot shows the current, the second plot shows the termination voltage, the third plot shows the superconductor voltage, and the bottom plot shows the inductive voltage of each cable (see Fig. 2).

The dynamic performance of the CORC^®^ triplet is simulated in Fig. 7 using the extracted parameters in Tables 1, 2, 3. A trapezoidal ramp to 3,900 A is simulated with a fast ramp rate of 10,000 A/s. The top plot shows the current, the second plot shows the termination voltage, the third plot shows the superconductor voltage, and the bottom plot shows the inductive voltage of each cable. With this fast ramp rate, inductive voltages drive over-critical current that can lead to cable damage. Fig. 7 shows an *L*/*R* decay at the constant current flat top (0.39–0.78 s) and the zero current region at the end of the ramp (1.17–1.56 s). In the zero current region (1.17–1.56 s), current is induced in the outer cables that oppose the direction of the transport current; this inductively driven current is a magnetization effect that is analogous to single ReBCO tapes^32,33^. At the constant current flat top (0.39–0.78 s), the *R* in the *L*/*R* decay consists of both flux-flow resistive voltage from the superconductor and termination resistances and, hence, the decay is quite rapid. At low current (1.17–1.56 s), only the termination resistances cause the induced current to decay and a longer time constant is observed.

### Quench detection via current redistribution monitoring

The quench detection scheme of Fig. 5 is shown in this section, which is based on the real-time differences between recreated and model-predicted cable currents for each incoming measurement. Both the magnitude of error and the rate of change of error are monitored using fixed error thresholds of 50 A and 250 A/s. As mentioned above, the number of false positive quench signals is greatly reduced by searching for signs of redistribution; in addition to a cable current decreasing until the error threshold is met, there must be an increase in current in the remaining cables.

**Figure 8:**
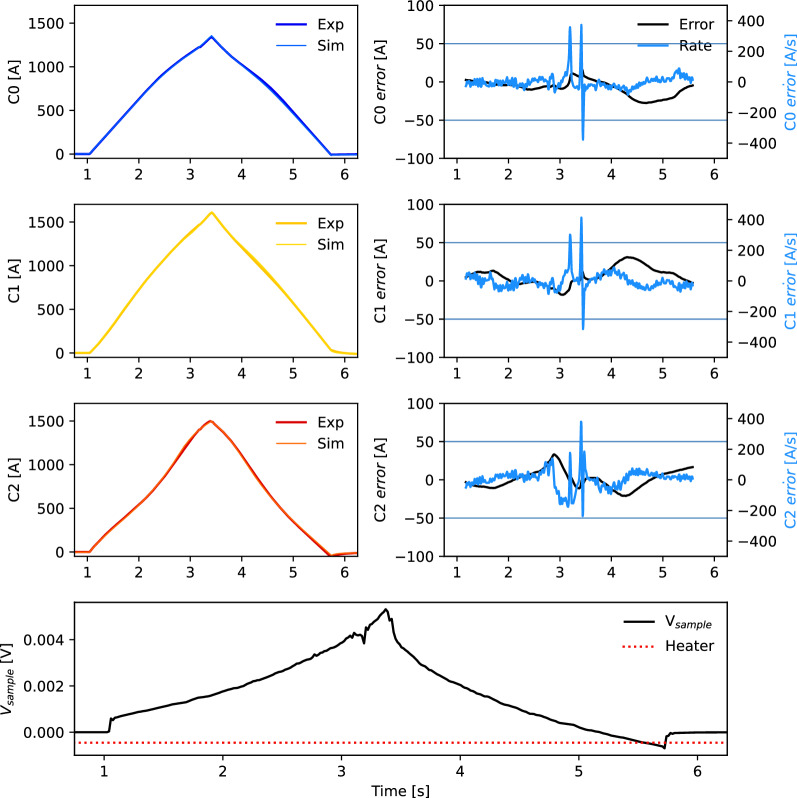
Current redistribution-based quench detection in CORC^®^ triplet test with dynamic ramp of 2000 A/s. No quench is induced in this case; this is correctly identified.

Figure 8 shows the triplet sample with no quench during a dynamic ramp of 2000 A/s to 3900 A where existing protection methodologies are less robust. All experiments are performed at 76 K. The top left plot (two blue curves) shows the inverse Biot-Savart recreated current and the model-predicted current in cable 0 (see Fig. 4), and the two plots below show central cable 1 (two yellow curves) and right cable 2 (two red curves). The right column shows the recreation error (black) and recreation error rate (blue) for each cable. The bottom plot shows the measured sample voltage (black) and quench heater (red). The error rates exceed the thresholds defined by the horizontal blue lines, even though no quench trigger (no vertical blue or black line) is produced; this demonstrates the effectiveness of a current redistribution-identifying algorithm, as all errors are in the same direction.

**Figure 9:**
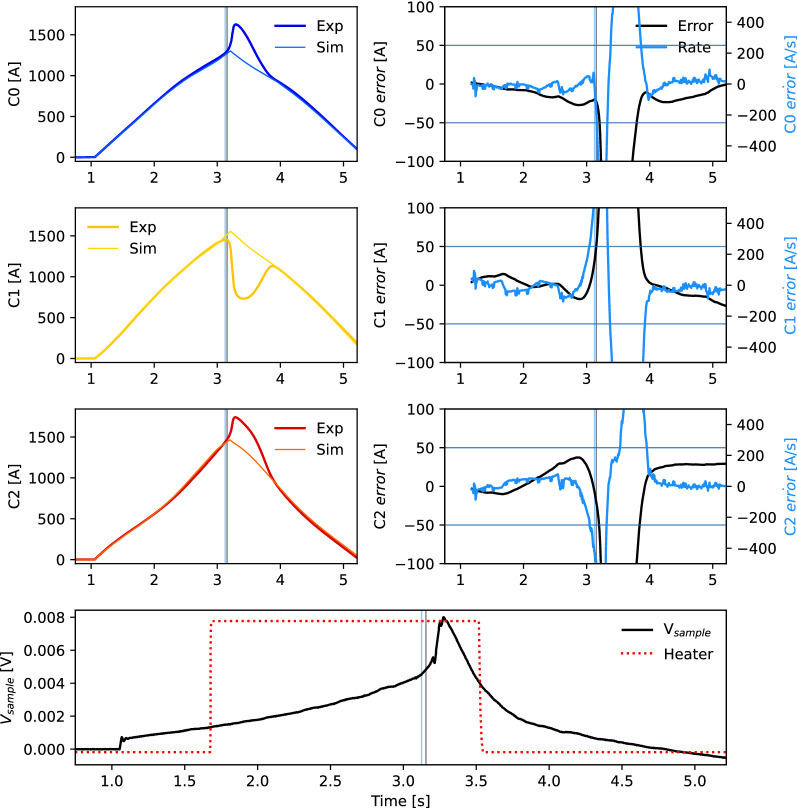
Current redistribution-based quench detection in CORC^®^ triplet test with dynamic ramp of 2000 A/s. Heater on C1 (middle cable) induces a quench that is correctly identified.

**Figure 10:**
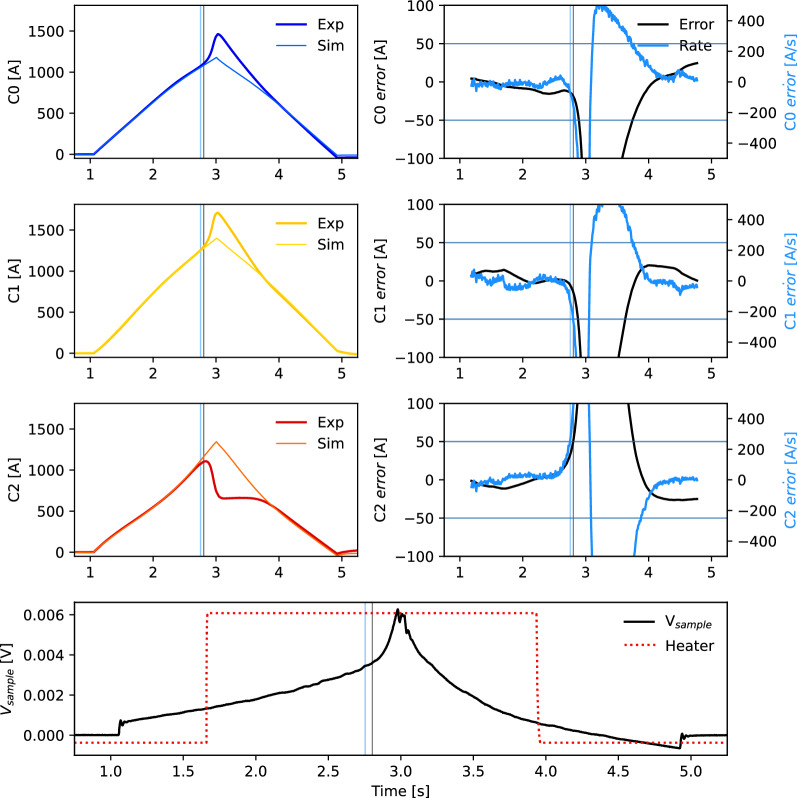
Current redistribution-based quench detection in CORC^®^ triplet test with dynamic ramp of 2000 A/s. Heater on C2 (right cable) induces a quench that is correctly identified.

Figures 9 and  10 show a similar 3900 A current ramp at 2000 A/s, however with heater induced quenches on C1 (middle cable) and C2 (right cable), respectively. The departure between experimental and simulated cable currents after firing the quench heater is shown in the right column, and the simulated quench trigger (vertical black, blue lines) is generated when the errors cross the thresholds (horizontal lines) and satisfy the redistribution criteria for five consecutive measurements. Note that the quenching cables have a different error direction, that is a signature of current redistribution.

## Discussion

The proposed methodology applied to the triplet sample in Ref.^26^ is shown to be a promising diagnostic for quality control, test planning and quench detection. There are downsides, as with any protection technique, and current distribution monitoring would likely supplement alternative detection methodologies such as voltage and temperature monitoring. In the limit of very large magnets with very fast ramp rates, inductive voltages will dominate resistive voltages from transitioning CORC^®^ cables and current redistribution will be minimal. In this limit, temperature monitoring may be a more viable quench detection technology^34^, however the current distribution monitoring methodology can still provide value in the quality control and test planning stages discussed earlier (Fig. 5). Having said this, Toroidal Field (TF) coils with demountable joints^4^ will consist of shorter coil segments with reduced inductance that will facilitate current distribution monitoring.

The inverse Biot-Savart procedure lies at the core of both the parameter extraction and real-time monitoring techniques, however this requires that cable current distributions are invariant of cable length. This applies to CICC with limited current sharing, such as the CORC^®^ 6-around-1^10,11^ where the inter-strand current sharing is a free design parameter that can be controlled by soldering CORC^®^ cables to a conductive support structure or by insulating CORC^®^ cables from each other. Care must be taken to position Hall probes such that the line current approximation is valid and single tape effects are minimized. It should be mentioned that alternative current sensing techniques are available. Although inter-tape current redistribution can be sensed in individual CORC^®^ cables^25^, it is more difficult to extract parameters and use a model to predict global current distributions based on measurements at the terminals.

A simplified electric circuit model may be required to protect magnets in real time without predefined current waveforms (i.e., coil currents are the output of a control loop^35,36^, that cannot be simulated in advance). If the magnet operating limit is redefined as the onset of superconducting voltage in any cable, the superconductor and resulting power law behavior can be removed from the circuit simulation (see Fig. 2). This can then be efficiently solved as a matrix system using finite difference approximations and measurements of the cable transport current. We prototyped this simplified dynamic network simulation on an inexpensive microcontroller with an ARM Cortex M7 processor, and the simulation process (single time step) took a fraction of a millisecond for the cable in Fig. 4. Future work will explore accelerating the full network simulation. The inverse Biot-Savart process was also prototyped with the same microcontroller and cable configuration, and the current recreation process was found to take a fraction of a millisecond. This was enabled by calculating the inverse $A^TA^{'}$ (Eq. 5) in advance.

## Data Availability

All data generated or analyzed during this study are included in the supplementary information files of this manuscript.
